# miR-181a decelerates proliferation in cutaneous squamous cell carcinoma by targeting the proto-oncogene KRAS

**DOI:** 10.1371/journal.pone.0185028

**Published:** 2017-09-20

**Authors:** Johannes Neu, Piotr Jan Dziunycz, Andreas Dzung, Karine Lefort, Martin Falke, Rémy Denzler, Sandra Nicole Freiberger, Guergana Iotzova-Weiss, Aleksandar Kuzmanov, Mitchell Paul Levesque, Gian-Paolo Dotto, Günther Franz L. Hofbauer

**Affiliations:** 1 Department of Dermatology, University Hospital Zurich, Zurich, Switzerland; 2 Department of Biochemistry, University of Lausanne, Epalinges, Switzerland; 3 Institute of Molecular Cancer Research, University of Zurich, Zurich, Switzerland; 4 Department of Biology, ETH Zurich, Zurich, Switzerland; University of Catanzaro, ITALY

## Abstract

Cutaneous squamous cell carcinoma (SCC) is the second most common human skin cancer with a rapidly increasing incidence among the Caucasian population. Among the many regulators, responsible for cancer progression and growth, microRNAs (miRNA) are generally accepted as key players by now. In our current study we found that microRNA-181a (miR-181a) shows low abundance in SCC compared to normal epidermal skin. *In vitro*, miRNA downregulation in normal primary keratinocytes induced increased proliferation, while *in vivo* miR-181a downregulation in HaCaT normal keratinocytes showed tumor-like growth increase up to 50%. Inversely, upregulation of these miRNAs in cancer cells lead to reduced cellular proliferation and induction of apoptosis *in vitro*. An in vivo therapeutic model with induced miR-181a expression in SCC13 cancer cells reduced tumor formation in mice by 80%. Modulation of miR-181a levels showed an inverse correlation with the proto-oncogene *KRAS* both on mRNA and protein level by direct interaction. Knockdown of *KRAS* mimicked the anti-proliferative effects of miR-181a overexpression in patient-derived SCC cells and abolished the enhanced viability of HaCaT cells following miR-181a knockdown. Furthermore, phospho-*ERK* levels correlated with *KRAS* levels, suggesting that the observed effects were mediated via the MAPK signaling pathway. miR-181a seemed regulated during keratinocyte differentiation probably in order to amplify the tumor suppressive character of differentiation. Taken together, miR-181a plays a crucial tumor suppressive role in SCC by targeting KRAS and could be a promising candidate for a miRNA based therapy.

## Introduction

Cutaneous squamous cell carcinoma (SCC) is the second most common skin malignancy in the general population with a rapidly rising incidence among Caucasians. It typically arises from intraepithelial lesions like actinic keratosis on sun-damaged skin [1, 2]. Disturbed differentiation represents a hallmark of SCC reflected by a diverse pattern of differentiation markers like Filaggrin and Involucrin or various keratins [3]. About 50% of all SCCs carry p53 mutations and a typical UV signature presenting with cyclobutane pyrimidine dimers, making UV light a major SCC risk factor [4]. Organ transplant recipients (OTR), however, harbor a 65–250 fold increased SCC incidence compared to the general population due to continued immunosuppression [5]. Once SCC occurs, further SCC arising on sun-damaged skin are likely. Field-directed treatments like photodynamic therapy, imiquimod or ingenol mebutate are available, but all cause considerable inflammation and disfiguration [6, 7]. Here, future siRNA or microRNA based agents could prove beneficial by reverting a keratinocyte’s course towards regular differentiation and cell death without inflammation.

miRNAs are approximately 20 nucleotide-long non-coding RNA molecules binding to the 3' untranslated regions (UTR) of target mRNAs in a sequence-specific manner influencing translation and/or stability of the transcripts [8]. miRNA effectively play roles in almost all aspects of cancer biology, such as in proliferation, apoptosis, metastasis and angiogenesis (reviewed in [9]). A large screen of miRNA expression in SCC singled out miR-181a as downregulated in our SCC patient samples. We thus studied expression and functionality of miR-181a in primary patient-derived and various cell lines representing normal skin and SCC. Our findings indicate a crucial role for miR-181a in regulating keratinocyte proliferation mediated by KRAS interaction and MEK pathway inhibition, prompting us to validate these findings in a xenograft mouse model.

## Materials and methods

### Cell culture

HaCaT, SCC13 and HEK293T [10–12] were cultured in DMEM high-glucose supplemented with 10% FCS (Gibco). Gibco’s SMF Keratinocyte medium, supplemented with EGF and Bovine Pituitary Extract (BPE), was used for SCC12 [11]. Primary patient-derived cells were grown in CnT-07 medium (CellNTec). Several cells were cultivated in a humidified 5% CO_2_ atmosphere at 37°C.

Institutional board approval for the use of human tissue was granted by the local IRB (Kanton of Zürich); all donors signed written informed consent forms in accordance with the Code of Ethics of the World Medical Association (Declaration of Helsinki) for experiments involving humans (ethical approval number EK647 and EK800). All samples were obtained from the University Hospital Zurich (Zurich, Switzerland).

### Viability and proliferation assays

2000–3000 treated cells/well were seeded into 96 well plates in the according growth medium. After 96 hours’ incubation the medium was swapped with fresh one containing 1:10 WST-1 (Roche) and 10% FCS. WST-1 assay was performed following the manufacturer’s protocol. Proliferation was assessed by bromodeoxyuridine (BrdU) incorporation using a BrdU assay Kit (Merck Millipore).

### RNA isolation and qPCR

RNA was isolated form pelleted cells or tissue samples using TRIzol (Invitrogen) following the manufacturer’s protocol. Promega’s Reverse Transcription System was used to transcribe mRNA samples into (poly T-) cDNA.

Gene expression analyses of coding genes (i.e. long transcripts) was performed using SYBR Green (Roche) while short transcripts (i.e. miRNAs) were analyzed using LifeTechnologies’ TaqMan microRNA Assays and small nuclear RNA Z30 as a housekeeping gene. All assays were performed according to the manufacturer’s protocols and quantified using ddCT method.

Primer sequences for SYBR green qPCR: 36B4 (housekeeping gene) forward: 3’—GCAATGTTGCCAGTGTCT—5’ reverse: 3’—GCCTTGACCTTTTCAGCA—5’, HRAS forward: 3’—GACGTGCCTGTTGGACATC- 5’ reverse: 3’–CTTCACCCGTTTGATCTGCTC—5’, KRAS forward: 3’—AAGACAGAGAGTGGAGGATGC—5’ reverse: 3’—GTGCTGAACTTAAACTTACCAGAT—5’, RhoA forward: 3’–GGAAAGCAGGTAGAGTTGGCT—5’ reverse: 3’—GGCTGTCGATGGAAAAACACAT—5’, pIND20 Notch1: forward: 3’—CCGCTACTCACGCTCTGA—5’ reverse: 3’—CTGGAAGACACCTTGTGC—5’, Involucrin: forward: 3’—TCCTCCAGTCAATACCCA—5’ reverse: 3’—CAGCAGTCATGTGCTTTT—5’, Filaggrin: forward: 3’—TGAAGCCTATGACACCAC—5’ reverse: 3’—TCCCCTACGCTTTCTTGT—5’, Loricrin: forward: 3’—GCGAAGGAGTTGGAG—5’ reverse: 3’—CTGGGTTGGGAGGTACT—5’: Keratin 10: forward: 3’—GGTGGGAGTTATGGAGGCAG—5’ reverse: 3’—CGAACTTTGTCCAAGTAGGAAGC—5’

### Protein detection

Cells were washed with PBS and lysed in RIPA buffer (Cell Signaling Technology, #9806). Protein extracts were analyzed by Western blotting using antibodies: anti-pp42/44 (#9101), anti-p42/44 (#9102), anti-rabbitHRP (#7074) all from Cell Signaling Technology. Anti-KRAS (sc-30) anti-Filaggrin (sc-66192), anti-Involucrin (sc-21748), anti-Loricrin (sc-51130), anti-b-actin (sc-47778) from Santa Cruz Biotechnology and anti-RhoA (ab187027) from abcam. Anti-mouse IgG (926–68022 and 925–32212) anti-rabbit IgG (926–68023 and 925–32213) and anti-goat IgG (926–68024 and 925–32214) all from LI-COR. Rabbit anti-mouse IgG H&L HRP (ab6728). Proteins were detected by ECL on Hyperfilm (Amersham, GE Healthcare) or by LI-COR Odyssey Infrared scanning system.

### Flow cytometry

SCC13 cells, transfected with miR-181a mimics, were stained using BD Pharmigen’s FITC Annexin V Apoptosis Detection Kit I following the manufacturer’s protocol. UVB-irradiated cells were used as a positive control and to adjust proper gate setup. Measurements were performed on a FACSCanto device (BD Biosciences). Data were analyzed with FlowJo software (Ashland).

### Transfection

Transient transfection of siRNAs (Qiagen), miRNA mimics, miRNA inhibitors and according control sequences (Abcam) was performed using Interferin (Polyplus). Larger constructs, like DNA plasmids, were transfected by the aid of GeneCarrier 1 (Epoch Biolabs). All transfections were carried out for 48 hours and following the manufacturer’s protocol. In short, cells were seeded 24 hours prior to transfection followed by medium change. The according transfection reagent was diluted in basal medium after nucleotides were added. After 30 minutes of incubation the solution was added drop wise to the cells and incubated at 37°C for 48 hours.

siRNA sequences: control sequence: 5’–AATTCTCCGAACGTGTCACGT– 3’, siKRAS_7: 5’–CTCCTAATTATTGTAATGTAA– 3’, siKRAS_8: 5’–AAGGAGAATTTAATAAAGATA– 3’.

### Transduction

Lentiviral particles were either obtained by Sigma Aldrich (MISSION® Lenti microRNA Inhibitors, MISSION® Lenti microRNA and the according control constructs) or produced using the psPAX2 Second Generation System in HEK293T cells. A detailed protocol for lentiviral particle production can be found in Barde et al [13]. In short, psPAX2 plasmids and the construct of interest were co-transfected into HEK293T cells using GeneCarrier-1 (Epoch Biolabs) for 24 hours, followed by daily medium change for 3 days in total. All supernatants were collected and pelleted in an ultra-centrifuge. Pellets were resuspended in DMEM 10% FCS and stored at -80°C. SCC13 cells were seeded 24 hours prior to the experiment. For the transduction, medium was swapped with DMEM 10% FCS containing 8μg/ml of hexadimethrine bromide (Sigma) and resuspended lentiviral particles overnight. The next day, medium was swapped with regular DMEM 10% FCS. After another 24 hours, the selection process was initiated using puromycin (Santa Cruz Biotechnology). Remaining cell clones were expanded and tested for functionality.

### *In vivo* tumor xenograft

Female nude mice (4–6 weeks old) were ordered from *Harlan Laboratories Inc*. All experiments were performed according to guidelines of the approved BLV protocol (Bew 104/2013, approved by the veterinary office of the Kanton Zürich). Furthermore, the veterinary office of the Kanton Zürich specifically approved the present study as an extension to our current license (license number: 24156).

4 × 10^6^ HaCaT kd miR-181a, 1 × 10^6^ of SCC13 Tet-On miR-181a, or the according number of control cells were suspended in PBS and injected subcutaneously after mice accommodated to their new habitat for 10–14 days. 10 animals per group were used for experiments involving HaCaT cells and 15 animals per group were used for *in vivo* SCC13 experiments. Doxycycline for the Tet-On experiment was administered via food pellets (200mg/kg) (Bioserv). The tumors were measured using a caliper three times a week. The tumor volume was calculated using the formula volume, V = L × W^2^/2, where L represents long diameter and W stands for short diameter. In parallel, mice where weighted once per week using a standard laboratory scale. Mice were euthanatized in case one or more termination criteria were fulfilled, such as tumor mass greater than 1cm^3^, ulcerating tumors, severe weight loss or lack of fight/flee instincts or at the experiment’s endpoint using CO_2_. Tumors of dead mice were bisected, whereat RNA was isolated from one half and Hematoxylin-eosin (H&E) slides were produced from the other part.

### Histology

Excised tumor from terminated mice were fixed in 4% formalin, followed by dehydration and embedding in paraffin. Seven micron sections were cut and stained with hematoxylin and eosin staining (H&E).

### Construction of Tet-On miRNA over expression plasmids

miR-181a hair pin sequence plus 210 base pairs of the flanking region in either direction and the according Gibson Assembly adapters were synthesized (Integrated DNA Technologies). The inserts were cloned into digested pTRIPZ (EcoRI and XhoI) using NEB’s Gibson Assembly kit following the manufacturer’s protocol. Empty pTRIPZ constructs served as an internal control.

### Generation of cell lines stably overexpressing KRAS

pUNO KRAS over expression plasmid (Invivogen) was transfected into cell lines as described above. Positive cells were selected using Blasticidin (5μM) (Invivogen). Clones were picked, expanded and checked for stable KRAS expression. For KRAS 3’UTR experiments pGL4.75-KRAS LCS6m (Addgene Plasmid #44571) was used as a KRAS 3’UTR donor and sub cloned into pUNO KRAS with NheI-HF and T4 DNA ligase (NEB). Three miR-181a sites were mutated using Phusion-HF polymerase for mutagenesis (NEB) and the following primers: First site: forward: 5’—CTTCTTATTTTTCTTACCAATTGTTCCGGTTGGTGTGAAACAAATTAATGAAGC—3’ reverse: 5’—GCTTCATTAATTTGTTTCACACCAACCGGAACAATTGGTAAGAAAAATAAGAAG—3’, second site: forward:

5’—GTCCTATAGTTTGTCATCCCTGATTCCGGTAAAGTTACACTGTTCACAAAGGTTTTGTC—3’ reverse: 5’—GACAAAACCTTTGTGAACAGTGTAACTTTACCGGAATCAGGGATGACAAACTATAGGAC—3’, third site: forward: 5’—CGTATATTGTATCATTTGAGTTCCGGTTCCCAAGTAGGCATTCTAGGC—3’ reverse: 5’—GCCTAGAATGCCTACTTGGGAACCGGAACTCAAATGATACAATATACG—3’.

The pUNO1 control plasmid (Invivogen) was used as a negative control during functional experiments

### MEK inhibitor

For MEK inhibition GSK1120212 (Cellagen Technology, #C4112-5) was used at concentrations of 500 nmol/L. DMSO (Sigma) was used to prepare stock solutions and also as a control treatment during the experiments.

### Ras family mutation analysis of cell lines

Mutation analysis of KRAS, NRAS and HRAS were performed strictly following the protocol by Chang and colleagues [14] 19879255. In short, genomic DNA from cell lines was isolated and purified using Qiagen’s DNA isolation kit (Cat No./ID: 51104). Regions of interest were PCR amplified followed by a gel purification step. Sanger sequencing was outsourced to Microsynth AG.

### Statistics

All statistical evaluations were carried out using GraphPad Prism 5.0. The analyses were two-tailed Student’s t-Test of three independent experiments. The error bars represent standard deviations (SD). In vivo experiments were evaluated using ANOVA with Bonferroni correction where error bars represent standard error of the mean (SEM). p-values of < 0.05 were considered significant.

## Results

### miR-181a is downregulated in SCC compared to normal skin

In order to get a general overview into the miRNA landscape in SCC we extracted total RNA of the epidermal fraction of SCC from patients (n = 15) as well as normal skin control samples (n = 5).

**Fig 1:**
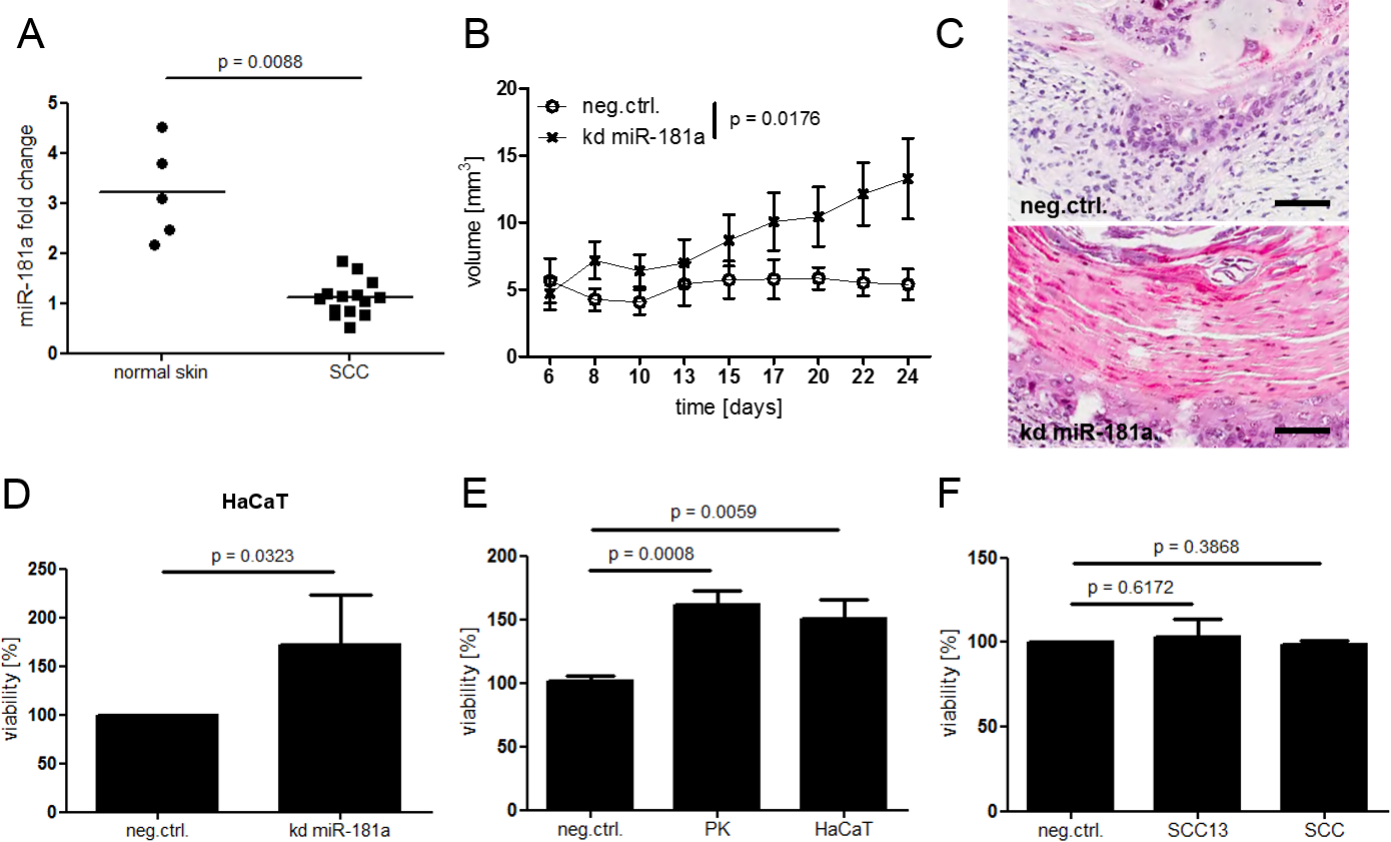
Low abundance of miR-181a is found in patient derived SCC specimen compared to normal skin and down regulation of miR-181a leads to increased viability in healthy keratinocytes. (A) Total RNA was isolated from patient derived SCC or normal skin biopsies (epidermal part). miR-181a levels were determined via TaqMan qPCR. (B) HaCaT knock down (kd) miR-181a or control cells were injected subcutaneously into nude mice. Over all significance of the time course experiment was calculated using two way Anova and Bonferroni correction. A Student’s t-Test was performed at the end point (day 24) (Figure B in S4 Fig). (C) H&E sections of cysts. Black bar = 100μm. (D) Viability of HaCaT kd miR-181a cells *in vitro*. (E + F) Cells were transfected with miR-181a inhibitors for 48 hours. For *in vitro* viability assays cells were seeded into 96 well plates and incubated for 96 hours followed by WST-1 assay. P values for in vitro assays were Calculated via Student’s T test. SCC = cultured SCC cells derived from patient samples, PK = cultured healthy keratinocytes derived from patient samples.

The initial microRNA microarray screen revealed a highly deregulated miRNA pattern of the two SCC groups when compared to normal skin. miR-181a showed low abundance among SCC confirmed by TaqMan qPCR (Fig 1A).

### Low levels of miR-181a result in increased cellular viability *in vivo* and *in vitro*

Decreased cellular presence of miRNAs presumably permits increased activity of their downstream targets and might in turn promote tumor formation. For the following functional assays, we set up a keratinocyte culture system from patient-derived SCC and normal cutaneous primary keratinocytes. In addition, we selected a panel of keratinocyte cell lines according to their basal miRNA expression levels (S1 Fig). Since HaCaT, a cell line derived from normal human keratinocytes, and human SCC cell lines SCC13 and A431 exhibit miR-181a levels similar to patient samples, these cell lines were considered most suitable for our experiments. With regard to subsequent functional assays we performed genotyping of H-, K-, and NRAS codons 12, 13, and 61 mutations in the HaCaT and SCC-13 cell lines. With the exemption of a heterozygous HRAS G12N mutation in the SCC-13 cell line, the two cell lines did not harbor genetic alterations in the sequenced regions (S2 Fig).

Indeed, HaCaT cells harboring a stable miR-181a knockdown (kd miR-181a) and injected subcutaneously into nude mice had pronounced cyst formation capability while the respective control cells hardly formed a cyst (Fig 1B and S4A and S4B Fig). H&E histological sections of the kd miR-181a cysts did not only differ in size, but revealed pathological characteristics typical for intraepithelial SCC: Disturbed differentiation with distinct eosinophilic hyperkeratosis accompanied by parakeratosis akin to human SCC. Control cysts, on the other hand, showed relatively inconspicuous keratinocyte differentiation and basophilic cornification (Fig 1C). *In vitro* WST-1 assays confirmed the inverse correlation between cellular viability and miR-181a levels; kd miR-181a showed greater values compared to control cells (Fig 1D). HaCaT kd miR-181a cells isolated from cysts after the experiments endpoint or from *in vitro* cultures exhibited lower miR-181a levels when compared to control cells (S4C Fig and S5 Fig). On the contrary, knockdown of miR-181a did not induce proliferation in primary SCC cells or SCC13 in which miR-181a was already lower, indicating that a 3-5-fold reduction in miR-181a was already sufficient to confer the maximum induction of proliferation (Fig 1F).

Due to its simplicity combined with a high degree of sensitivity as well as reliability, WST-1 is widely used to investigate differences in cellular proliferation rates. We are well aware that WST-1 is reduced in the mitochondria during events happening in the respiratory chain and reflects therefore metabolic activity. To evaluate this assay for our needs, we performed a WST-1 assay on healthy primary keratinocytes transfected with miR-181a or control inhibitors. In parallel all cells were counted at the starting and end point of the assay and normalized to control cells. In addition, we performed a BrdU incorporation assay, which represents another widely used, but more complex proliferation assay. Since the WST-1 measurement, manual cell counting and BrdU incorporation assay delivered comparable results, we concluded that mitochondrial activity adequately reflects cellular proliferation rates in keratinocytes (S3 Fig). For the sake of correct scientific terminology however, we will stick to the term “viability” in our graphs.

### High levels of miR-181a result in decelerated cellular viability in vivo and in vitro

**Fig 2:**
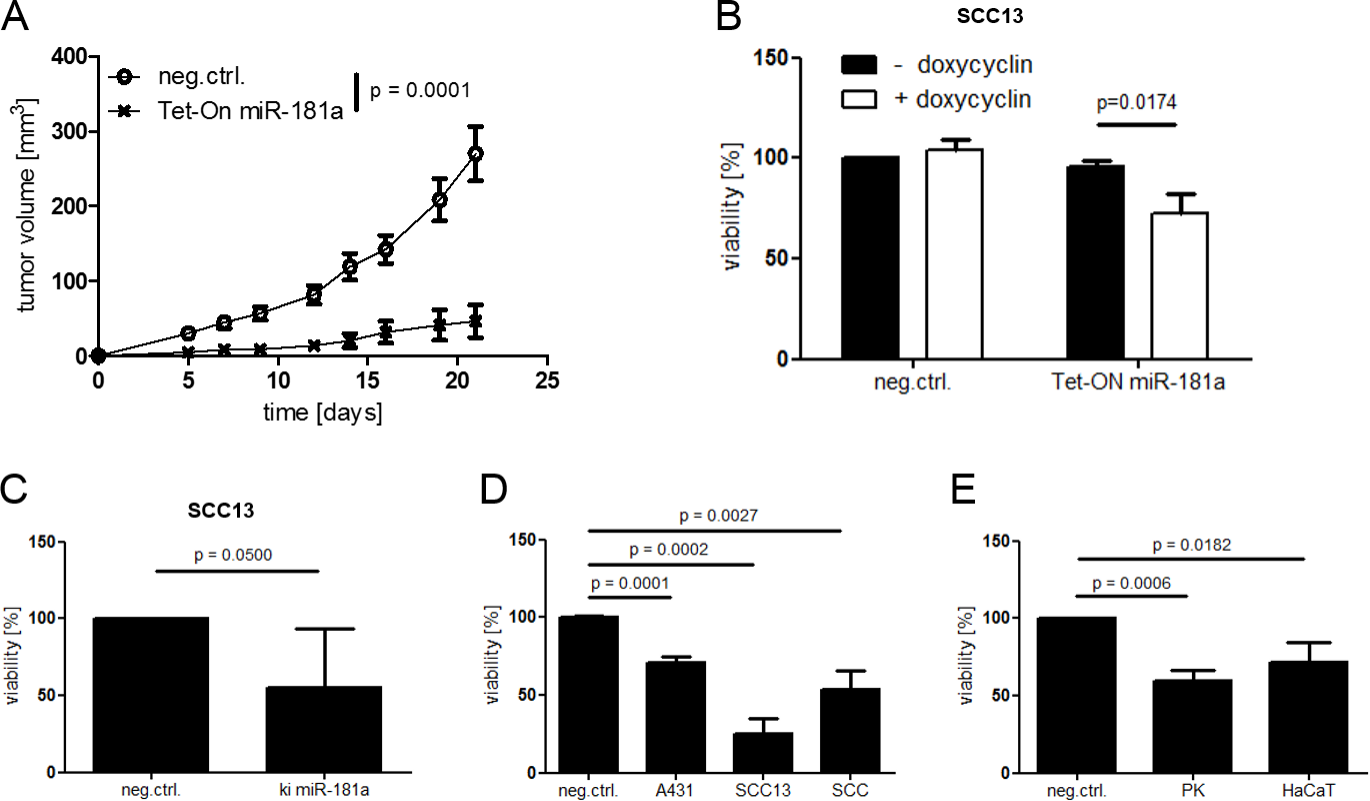
Up regulation of miR-181a leads to decreased viability in cancer cells. (A) SCC13 was transduced with pTRIPZ for inducible miRNA overexpression (SCC13 Tet-ON miR-181a) and injected subcutaneously into nude mice. Doxycycline (200 mg/kg) was administered via food pellets. Over all significance of the time course experiment was calculated using two way Anova and Bonferroni correction. (B) SCC13 Tet-On miR-181a were seeded into 96 well plates and exposed to doxycycline during the course of the experiment. Cells were transduced with pLKO.miRNA (ki miR-181a) for stable miRNA overexpression (C) or transfected with miRNA mimics for 48 hours (D + E). Cells were seeded into 96 well plates and incubated for 96 hours followed by WST-1 viability assay. P values for *in vitro* assays were calculated via Student’s t-Test. SCC = cultured SCC cells derived from patient samples, PK = cultured healthy keratinocytes derived from patient samples.

Following our observation of an inverse correlation between miR-181a levels and cellular viability, we were speculating that an upregulation of miR-181a would lead to decreased cellular viability in cancer cells. Therefore, we established a tetracycline-inducible miR-181a over expression model based on pTRIPZ Tet-On plasmid and SCC13 cells (Tet-On miR-181a). When injected subcutaneously into nude mice, control cells formed rapidly growing tumors, reaching termination criteria (tumor size > 1cm^3^ and/or ulceration) relatively early. miR-181a over expressing cells, on the other hand, grew slower and reach termination criteria at later time points (Fig 2A and Figures A-C in S6 Fig).

In addition, Tet-On miR-181a showed decreased viability when doxycycline was added to the medium (Fig 2B). Maintained miR-181a expression in tumors with Tet-On miR-181a and in *vitro cell* cultures confirmed a robust miRNA induction by doxycycline activation (S6D Fig and S7A Fig). Fittingly, transfection of synthetic miRNAs (miRNA mimics) into cancer cells or stable miR-181a knock in led to decreased cellular viability accompanied by cell rounding and detachment (Fig 2D and Figure B in S8 Fig). FACS analyses as well as immunoblotting revealed a high number of apoptotic cells, identified by AnnexinV+/7AAD- staining and Caspase-3 cleavage respectively, compared to the control group transfected with control miRNA (Figure A in S8 Fig). Interestingly, healthy primary keratinocytes and HaCaT cells were vulnerable to miR-181a upregulation to a certain degree as well (Fig 2E). An additional conformation was done in SCC13 cells transduced with a stable miR-181a over expression plasmid (ki miR-181a). As expected, these cells exhibited lower viability compared to control cells, while their miR-181a levels were strongly upregulated (Fig 2C and Figure B in S7 Fig).

### *KRAS* is a direct target of miR-181a

Next we aimed to unravel the molecular mechanism laying behind miR-181a’s negative effect on cellular viability. The proto-oncogene *KRAS* plays a critical role in a variety of malignancies and the interplay of miR-181a and *KRAS* has been described in other epithelial cancers [15].

**Fig 3:**
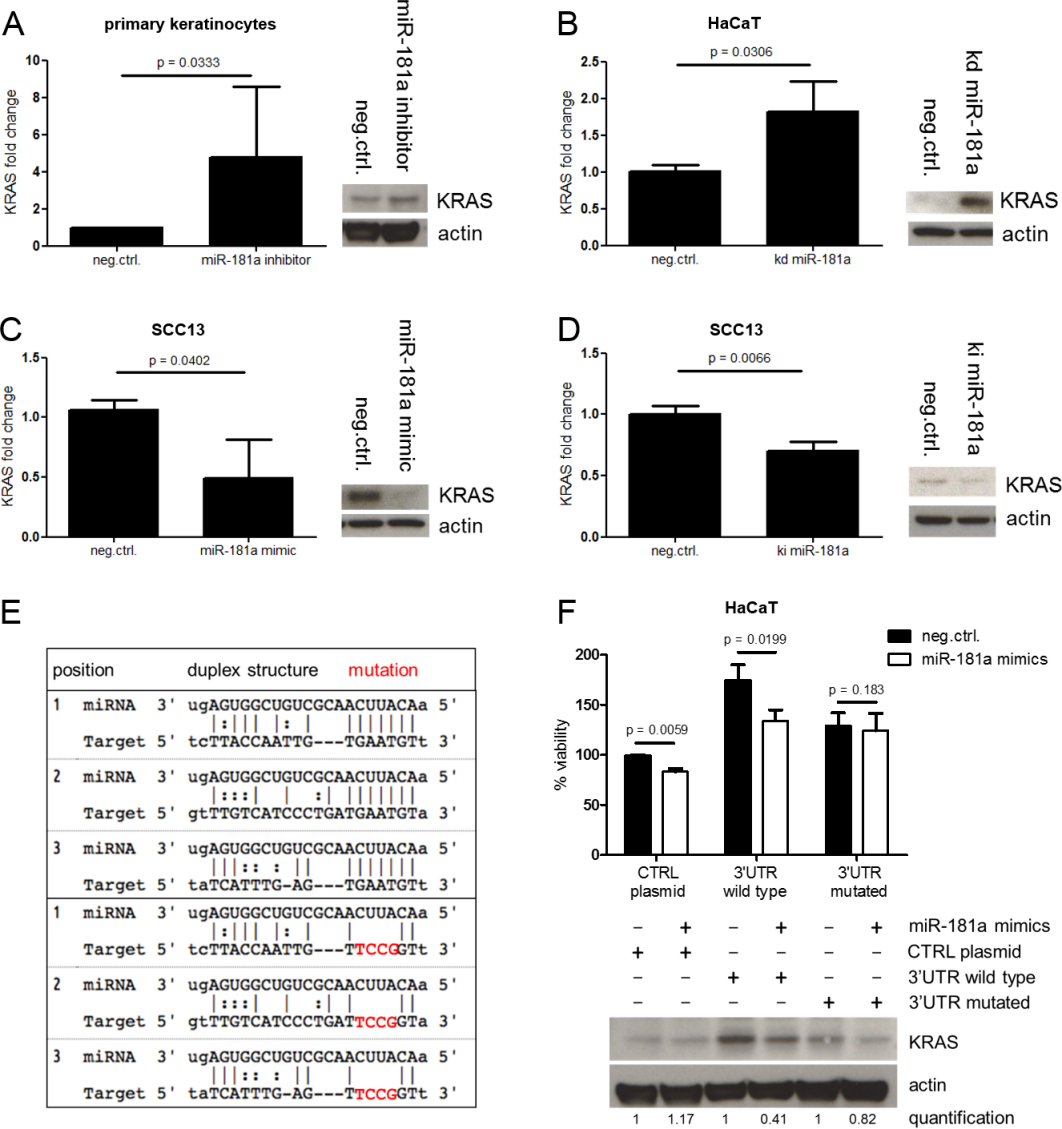
miR-181a targets KRAS directly. Cells were transfected with miRNA mimics (A) or inhibitors (C) for 48 hours or transduced with pLKO.miRNA.inhibitor / pLKO.miRNA (kd miR-181a / ki miR-181a) for stable miRNA modulation (B and D). Cells were lysed and mRNA or protein level analyses were performed using SYBR green qPCR or Western Blot respectively. (F) HaCaT cells were stably transfected with the indicated pUNO KRAS over expression plasmids in selection medium. Selected clones were transfected with miR-181a mimics for 48 hours following by WST-1 assay and Western Blot. (E) The panel in the left illustrates the three main miR-181a binding sites in KRAS 3’UTR and the according mutations. Student’s t-Test was used to calculate P values.

Healthy cells with repressed miR-181a, either by transfection of miRNA inhibitors (Fig 3A) or by stable knock down (Fig 3B), showed higher *KRAS* protein and mRNA levels. SCC13 cells, on the other hand, exhibited lower *KRAS* levels when miR-181a was upregulated either by transfection of miRNA mimics (Fig 3C) or by stable miR-181a over expression (Fig 3D).

miRNAs are interacting with the 3’UTRs of their target mRNAs in a sequence-specific manner and are thereby interfering in the process of protein translation. In our approach we took advantage of the *KRAS* overexpression plasmid pUNO KRAS and cloned the according 3’UTR upstream of the start codon. A 3’UTR containing mutated miR-181a binding sites served as a control (Fig 3E). HaCaT cells stably transfected with these plasmids were characterized by elevated *KRAS* protein levels. However, only the cells containing the wild type *KRAS* 3’UTR were vulnerable to miR-181a mimic transfection resulting in reduced *KRAS* protein translation and thus in reduced cellular viability (Fig 3F). The same *KRAS* 3’UTR system transfected into HEK293T cells showed a similar picture. Cells harboring the wild type *KRAS* ‘3UTR had reduced *KRAS* protein after miR-181a mimics treatment while cells harboring a mutated 3’UTR were less responsive to miR-181a mimics (S9 Fig).

Notably, the Ras family members *HRAS* and *RhoA* are most likely not subjected to miR-181a posttranscriptional regulation in SCC13 cells as demonstrated by transfectional miR-181a modulation and gene expression analysis via qPCR and Western blotting (S12 Fig).

### miR-181a mediates its tumor-suppressive role through *KRAS* signaling via the MAPK pathway

**Fig 4:**
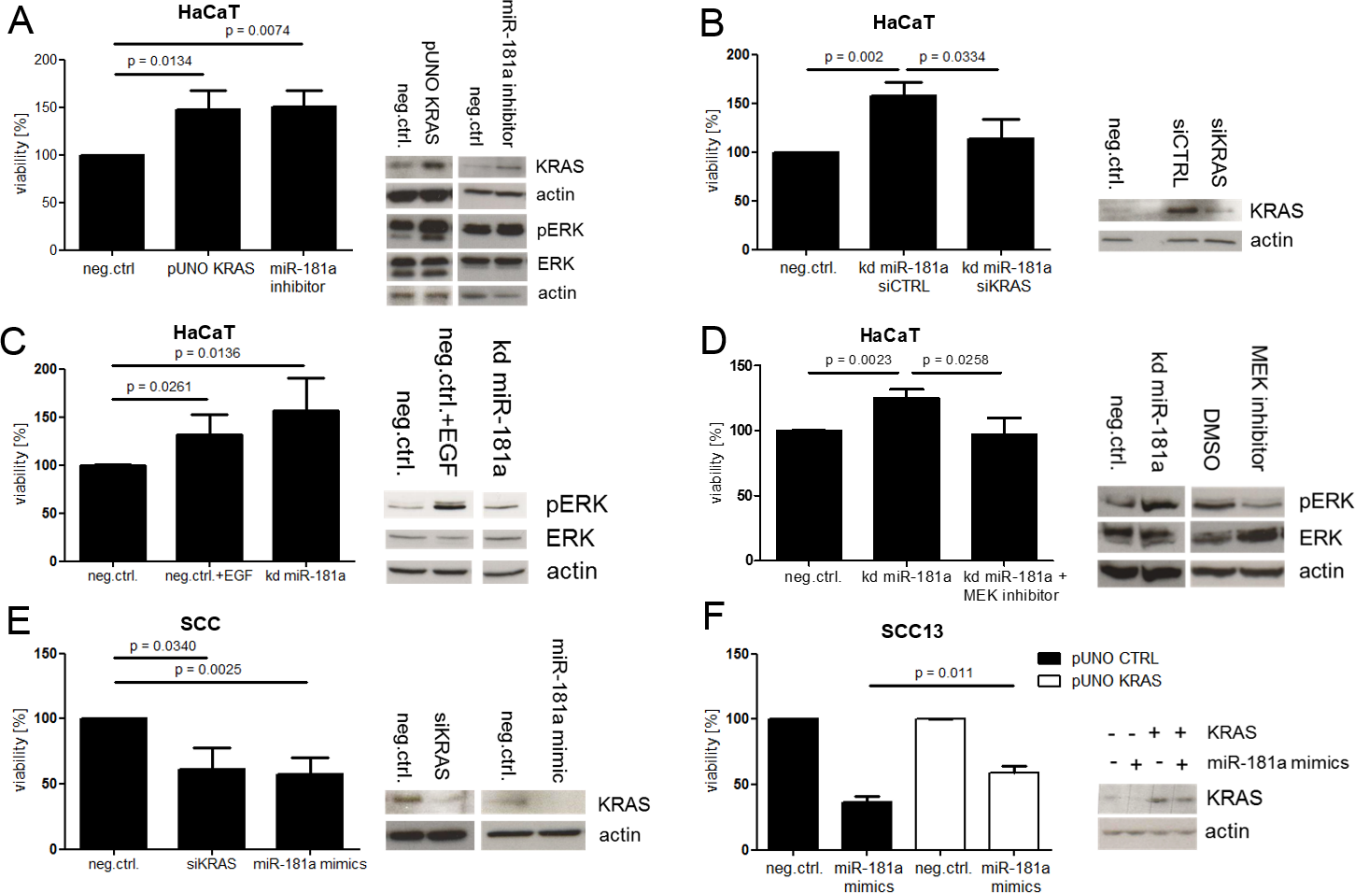
miR-181a mediates its tumor suppressive role through KRAS which signals via the MAPK pathway. Cells were transfected with the indicated siRNAs or miRNA mimics (A). In addition to siRNA/mimics the cells were transduced with the indicated plasmids (A—F). All transfections were carried out for 48 hours. A subset of the cells was seeded into 96 well plates, followed by WST-1 assay, while RNA and protein was isolated from the left overs. Protein and mRNA levels were determined by Western Blot or SYBR Green qPCR respectively. Student’s t-Test was used to calculate P values. kd = knock down, SCC = cultured SCC cells derived from patient samples.

Following the observation that miR-181a in keratinocytes correlated negatively with *KRAS* expression and with cellular viability, we were interested in a functional role of KRAS in keratinocytes. First we stably overexpressed *KRAS* in HaCaT using the pUNO *KRAS* plasmid (HaCaT pUNO KRAS) and performed a WST-1 assay. The result revealed that *KRAS*, when overexpressed, was able to boost viability in HaCaT similarly as a miR-181a knock down did. Notably, MAPK signaling was activated during this process (Fig 4A). Aiming to address the question whether there is a direct functional connection between miR-181a and *KRAS* we transfected HaCaT kd miR-181a with siRNA against *KRAS* or control siRNA. As expected, HaCaT cells harboring a stable miR-181a knock-down proliferated faster when only transfected with control siRNA, similarly as observed before. Reducing miR-181a’s target *KRAS*, using siRNA, abolished this effect (Fig 4B). As mentioned before, MAPK signaling was activated as a result of *KRAS* overexpression. Therefore, we speculated that *KRAS* uses the MAPK signaling pathway to mediate its oncogenic signals in the cell. Initially, we treated HaCaT cells using epidermal growth factor (EGF) to trigger MAPK signaling, resulting in enhanced proliferation rates similarly as observed in HaCaT kd miR-181a (Fig 4C). Furthermore, we used HaCaT kd miR-181a which exhibited increased viability compared to control. This difference was abolished upon treatment with a *MEK* inhibitor (Fig 4D).

Suppression of *KRAS* by siRNA knock down, on the other hand, decreased viability in primary patient-derived SCC cells (SCC) similarly as miR-181a overexpression did (Fig 4E). Additionally, overexpressed *KRAS* rescued miR-181a mimics-induced effects in SCC13 to a large part (Fig 4F).

### miR-181a levels increased during keratinocyte differentiation

Since SCC can be characterized by disturbed differentiation, we speculated that miR-181a expression might be regulated during this process.

First we differentiated primary healthy keratinocytes by keeping them cultured confluent for a week. Direct cell-cell contact is required for Notch1 activation which subsequently initiates the differentiation cascade. Comparing these matured cells with proliferating ones revealed increased miR-181a levels accompanied by clearly elevated differentiation markers in the differentiated subset (Figure A in S10 Fig). SCC13 cells which cannot differentiate due to Notch1 defects, regained the ability to mature when Notch1 was inducibly knocked in. During differentiation miR-181a levels went up (S10C Fig and S11 Fig). Induction of keratinocyte differentiation by adding Ca^2+^ to the medium or by UVA irradiation lead to similar results (Figures B and D in S9 Fig).

## Discussion

In our study we have identified miR-181a as a critical factor in SCC progression. The published body of evidence on miR-181a mainly shows a carcinogenic role for miR-181a such as in [16–19], making miR-181a a so-called `oncomir´. A smaller part of the data on miR-181a, however, identifies miR-181a as a tumor suppressor in some cancers of the brain and the hematopoietic lineage [20–22]. This tumor suppression seems mainly to impact invasion and metastasis in tumors of the liver [23], salivary glands [24] and the ovary [25], and to a smaller part to impact proliferation such as in non-small cell lung cancer (NSCLC) [26] and acute myeloid leukemia [27]. These discrepancies on the function of miR-181a in different tumors, highlight miR-181a’s context specificity depending on the environment. Our data show a tumor-suppressive role of miR-181a in the context of keratinocyte cancer with an impact on SCC proliferation. Contrary to miR-181a knockdown, reestablishing miR-181a levels in SCC attenuates cancer both *in vivo* and *in vitro*. Showing functionality beyond epithelial cancers, miR181a overexpression suppresses cell growth in xenograft tumors in chronic myelogenous leukemia [28] and large B-cell lymphoma [29]. The effect size on SCC in our study seems larger, suggesting therapeutic potential for miR-181a against SCC.

Based on known interaction of miR181a with *KRAS* [30], our study identified a direct interaction of the proto-oncogene *KRAS* with miR-181a in SCC confirming findings from other organ tumors [15, 26]. While this direct interaction has already been demonstrated using a dual luciferase assay [15, 27], we document this interaction in our system in a novel fashion by mutating the 3’-UTR of *KRAS* coupled with actual *KRAS* expression. This system allowed us to perceive the miR-181a-*KRAS* interaction above the background of endogenous KRAS expression. We thus support the previously found interaction between miR-181a and KRAS 3’UTR and directly link its main functional consequence to SCC, namely a change in cellular viability. Furthermore, the protein levels of Ras family members *HRAS* and *RhoA* were not affected by miR-181a modulation further underlining *KRAS* as miR-181a’s functional target in SCC. The mechanism of *KRAS* in keratinocytes, however, is only sketchily described. Our data confirm that the well-known downstream mediators of *KRAS* function are found in the MAP kinase pathway [24, 31]. While *KRAS* acts as an oncogenic driver mainly by activating mutations [32–34], posttranscriptional control of *KRAS* by miRNA deregulation has been identified repeatedly [35–38]. Our data suggest that such posttranscriptional control of *KRAS* by miR-181a may be critical in SCC development.

Differentiation is a key mechanism in cell control, maintaining homeostasis and preventing cancer development [39, 40]. Our data shows that differentiation in keratinocytes at large increases miR-181a expression, suggesting a role for miR-181a in a common pathway of differentiation, and that removing miR-181a is critical for the transition of keratinocytes into SCC. A similar weight has been reported in keratinocytes for miR-203 and miR-24 [41–43], underlining the impact of miRNAs in differentiation [44–46]. We thus believe that miR-181a is an important mediator of differentiation in keratinocytes. In summary, our data singles out miR-181a as a critical determinant in keratinocyte differentiation and control of SCC development. Manipulating miR-181a in vivo demonstrates the potential of miR-181a as a potential therapeutic miRNA in SCC.

## Supporting information

S1 Fig Endogenous miR-181a levels of cell lines used in the present study. Total RNA was isolated and transcribed into cDNA. miR-181a levels of various cell lines were determined via TaqMan qPCR. PK = cultured healthy keratinocytes derived from patient samples, SCC = SCC cells derived from patient samples.
(TIF)

S2 Fig Genotyping of H-, K-, and NRAS codons 12, 13, and 61 mutations in keratinocyte cell lines. The used cell lines, HaCaT and SCC-13, were assessed for their mutation status in the activating codons (red arrows) of the HRAS (A+B), KRAS (C+D) and NRAS (E+F) genes. Genomic DNA was isolated and regions of interest were amplified using PCR. Products were gel purified and prepared for Sanger sequencing.
(TIF)

S3 Fig WST-1 viability assay represents keratinocyte proliferation rates. Primary keratinocytes and HaCaT cells were transfected with miR-181a inhibitors or control sequence and seeded into Petri dishes. Cells were incubated for 96 hours and manually counted at the experiment’s end point. WST-1 and BrdU assays were performed in 96 well plates after 96 hours of incubation time. Statistical analysis was performed using Student’s t-Test. PK = cultured healthy keratinocytes derived from patient samples.
(TIF)

S4 Fig miR-181a knock down promotes cyst formation *in vivo*. (A) HaCaT knock down (kd) miR-181a cells were injected subcutaneously into nude mice. Arrows in the upper panels highlight cysts or rudimental cysts. Lower panels show H&E stainings of the cysts. Length of black bars = 1mm, Length of green bars = 30 μm. (B) Cyst volumes at the experiment’s end point (day 24). (C) miR-181a levels of cysts isolated after mice were terminated. Whole RNA was isolated and transcribed into cDNA followed by TaqMan qPCR. Statistics were performed using Student’s t-Test and Welch’s correction. kd = knock down.
(TIF)

S5 Fig miR-181a levels of HaCaT cells harboring stable miR-181a knock down. Whole RNA was isolated and transcribed into cDNA. miR-181a levels of HaCaT kd miR-181a were determined via TaqMan qPCR. Statistics were performed using Student’s t-Test. kd = knock down.
(TIF)

S6 Fig Up regulation of miR-181a leads to decreased tumor growth in vivo. (A) SCC13 Tet-ON miR-181a were injected subcutaneously into nude mice. Doxycycline (200 mg/kg) was administered via food pellets. (B) Tumor volumes at the statistical endpoint of the experiment (day 21). (C) Kaplan-Meier survival curve. (D) miR-181a levels of tumors isolated after mice were terminated. Whole RNA was isolated and transcribed into cDNA follow by TaqMan qPCR. Statistics were performed using Student’s t-Test and Welch’s correction.
(TIF)

S7 Fig miR-181a levels of SCC13 with artificial miR-181a up regulation. (A) SCC13 Tet-On miR-181a were incubated with 500nM doxycycline for 48 hours. (B) SCC13 stably over expressing miR-181a. Whole RNA was isolated and transcribed into cDNA followed by TaqMan qPCR. Statistics were performed using Student’s t-Test. ki = knock in.
(TIF)

S8 Fig miR-181a up regulation induces apoptosis in SCC13. (A) SCC13 cells were transfected with miR-181a mimics for 48 hours. One fraction was stained for 7AAD and AnnexinV following FACS analysis. Cells were irradiated with 0.06 J/cm^2^ UVB for gating setup and served as a positive control. Protein from the other fraction was isolated and used for cleaved caspase 3 determination via Western blotting. (B). Cells were transfected with miR-181a mimics for 48 hours and seeded into petri dishes. 96 hours post transfection pictures were taken. Length of black bars = 150μm.
(TIF)

S9 Fig miR-181a targets KRAS directly. Indicated pUNO KRAS over expression plasmids and miR-181a mimics were transfected simultaneously into HEK293T cells for 48 hours. Protein was extracted followed by Western Blot. The panel in the left illustrates the three main miR-181a binding sites in KRAS 3’UTR and the according mutations.
(TIF)

S10 Fig miR-181a levels are increased during keratinocyte differentiation. Cells were differentiated by keeping them in a confluent state for a week (A), by Ca^2+^ exposure (B), by transducing an inducible Notch1 plasmid (C) or by exposing them to 25 joule UVA irradiation. Protein and mRNA levels were determined by Western Blot or qPCR respectively. Student’s t-Test was used to calculate P values.
(TIF)

S11 Fig SCC13 cells harboring a tetracycline inducible Notch1 construct. SCC13 pIND20 Notch1 were incubated with 500nM doxycycline for 48 hours. Whole RNA was isolated and transcribed into cDNA followed by TaqMan qPCR. qPCR primers were designed to target the expression sequence (intra-cellular domain of Notch1) of pIND20 Notch1.
(TIF)

S12 Fig miR-181a modulation does not lead to changes in mRNA/protein levels of Ras family members HRAS and RhoA. Cells were transfected with miRNA inhibitors (A) or mimics (B) for 48 hours. Cells were lysed and protein level analyses were performed using Western Blot. Total RNA extracts were used for oligo dT cDNA synthesis followed by SYBR Green qPCR. Error bars represent standard deviation and Student’s T test was used to calculate P values.
(TIF)
