# Supplementary figures and images for: miR-181a decelerates proliferation in cutaneous squamous cell carcinoma by targeting the proto-oncogene KRAS
Source: PLoS One. 2017 Sep 20;12(9):e0185028. doi: 10.1371/journal.pone.0185028 (PMC5607211; doi:10.1371/journal.pone.0185028)

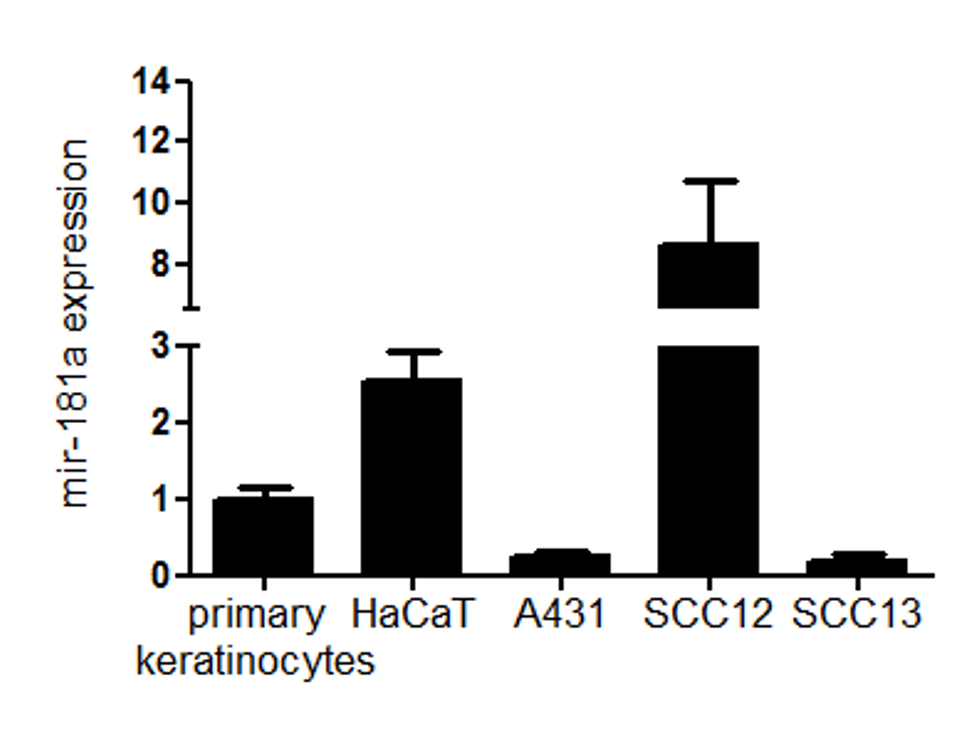

Supplement: S1 Fig — Total RNA was isolated and transcribed into cDNA. miR-181a levels of various cell lines were determined via TaqMan qPCR. PK = cultured healthy keratinocytes derived from patient samples, SCC = SCC cells derived from patient samples. (TIF) [file pone.0185028.s001.tif]

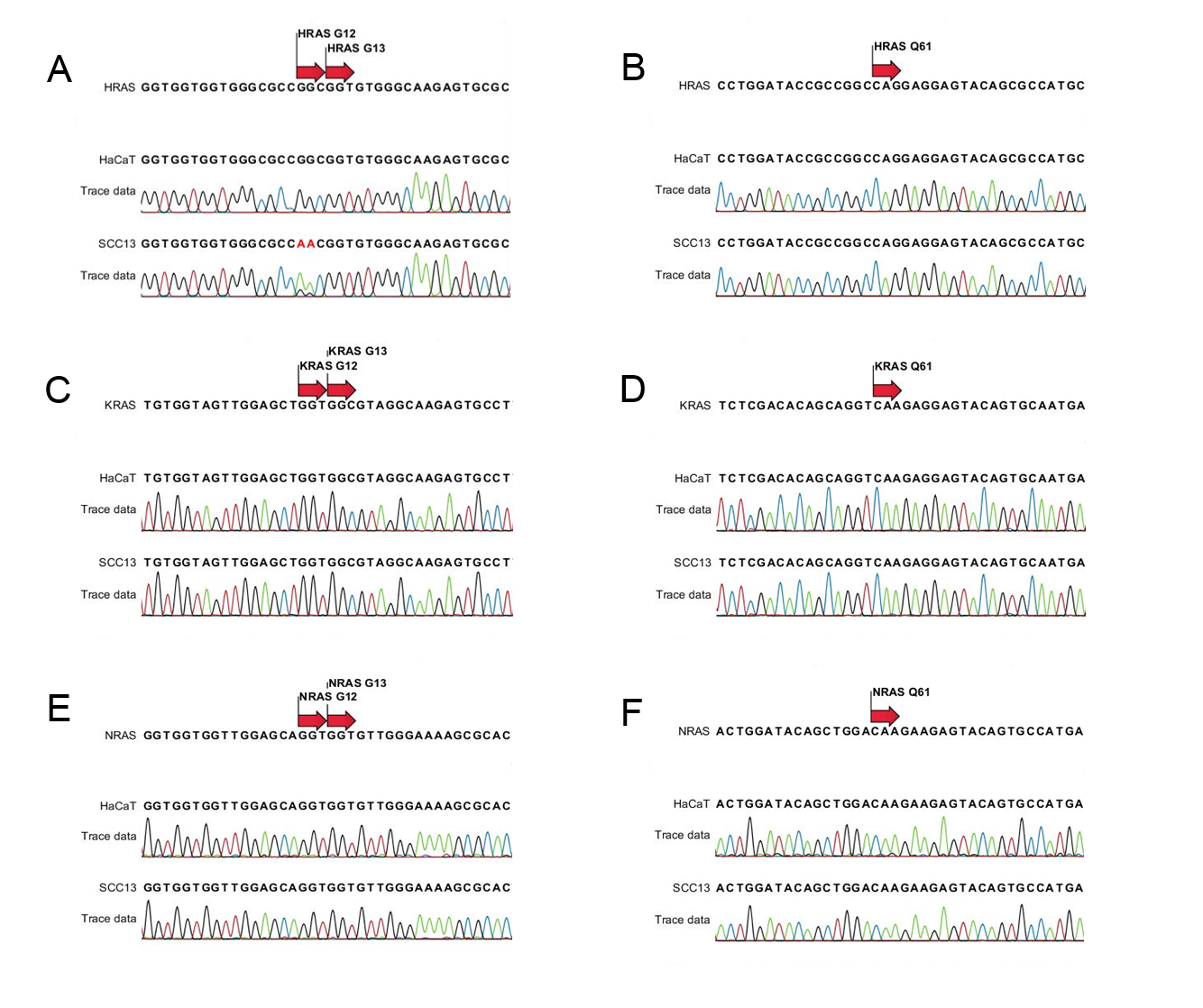

Supplement: S2 Fig — The used cell lines, HaCaT and SCC-13, were assessed for their mutation status in the activating codons (red arrows) of the HRAS (A+B), KRAS (C+D) and NRAS (E+F) genes. Genomic DNA was isolated and regions of interest were amplified using PCR. Products were gel purified and prepared for Sanger sequencing. (TIF) [file pone.0185028.s002.tif]

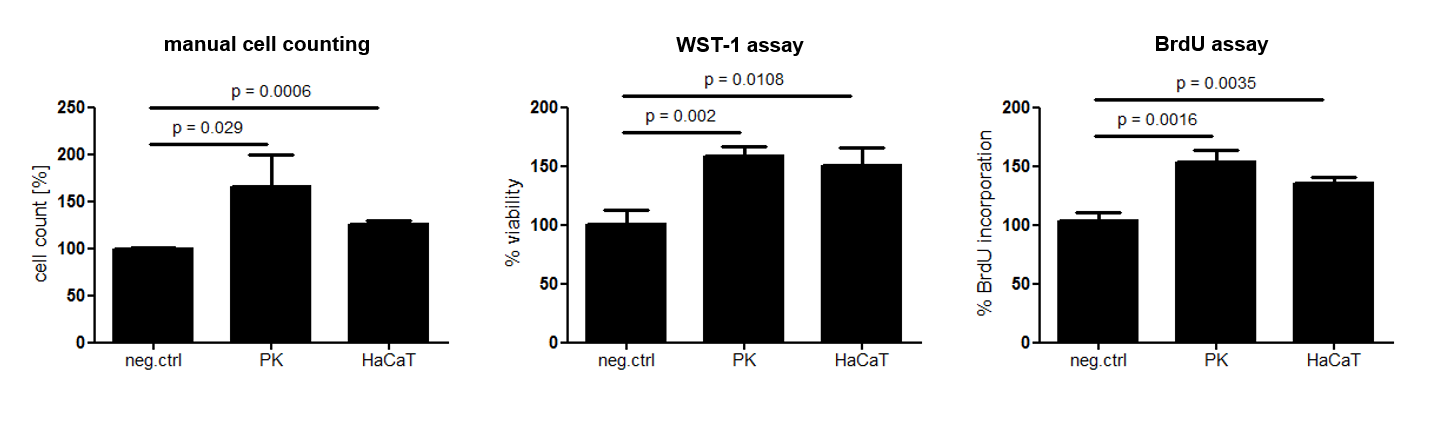

Supplement: S3 Fig — Primary keratinocytes and HaCaT cells were transfected with miR-181a inhibitors or control sequence and seeded into Petri dishes. Cells were incubated for 96 hours and manually counted at the experiment’s end point. WST-1 and BrdU assays were performed in 96 well plates after 96 hours of incubation time. Statistical analysis was performed using Student’s t-Test. PK = cultured healthy keratinocytes derived from patient samples. (TIF) [file pone.0185028.s003.tif]

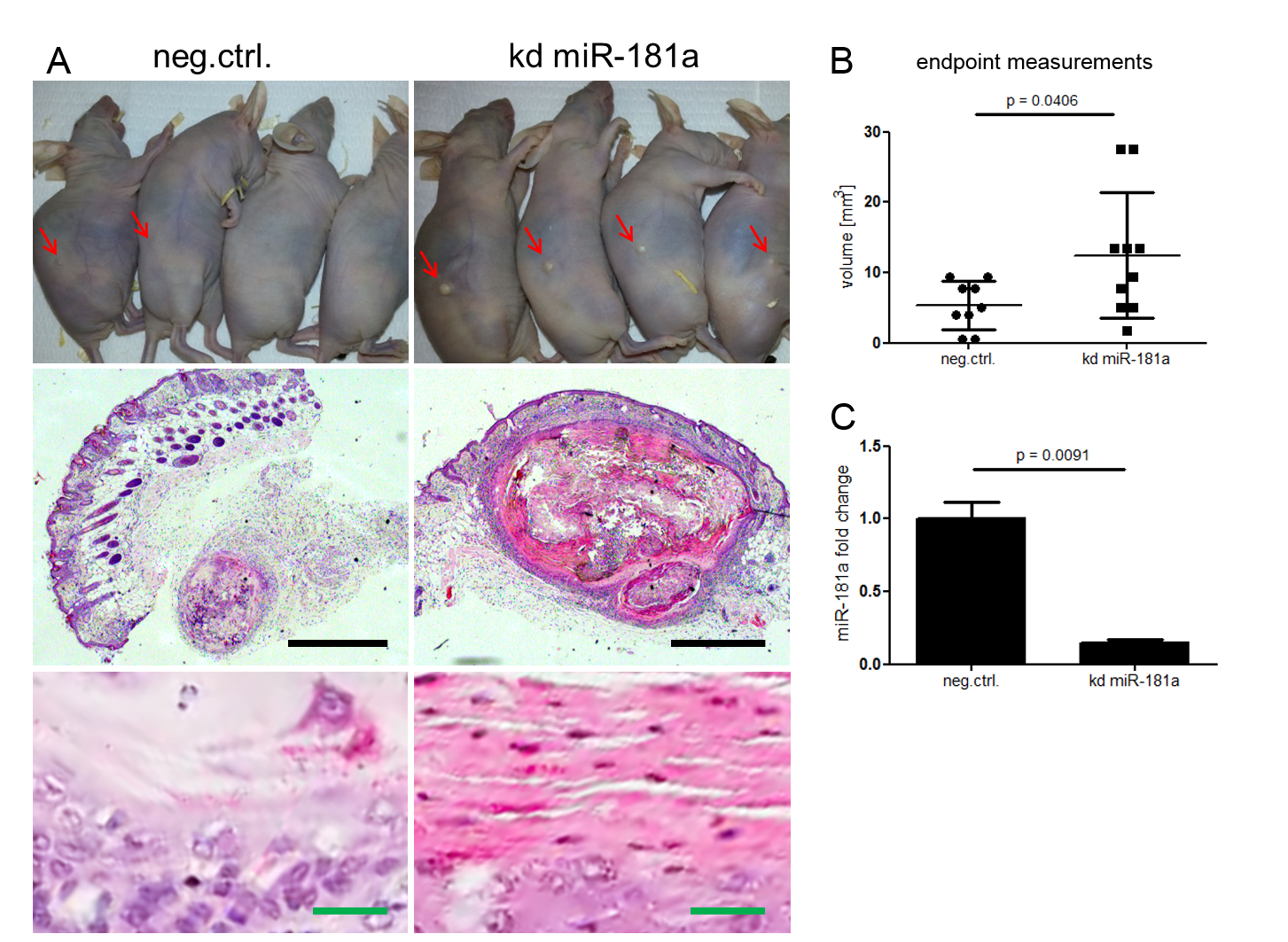

Supplement: S4 Fig — (A) HaCaT knock down (kd) miR-181a cells were injected subcutaneously into nude mice. Arrows in the upper panels highlight cysts or rudimental cysts. Lower panels show H&E stainings of the cysts. Length of black bars = 1mm, Length of green bars = 30 μm. (B) Cyst volumes at the experiment’s end point (day 24). (C) miR-181a levels of cysts isolated after mice were terminated. Whole RNA was isolated and transcribed into cDNA followed by TaqMan qPCR. Statistics were performed using Student’s t-Test and Welch’s correction. kd = knock down. (TIF) [file pone.0185028.s004.tif]

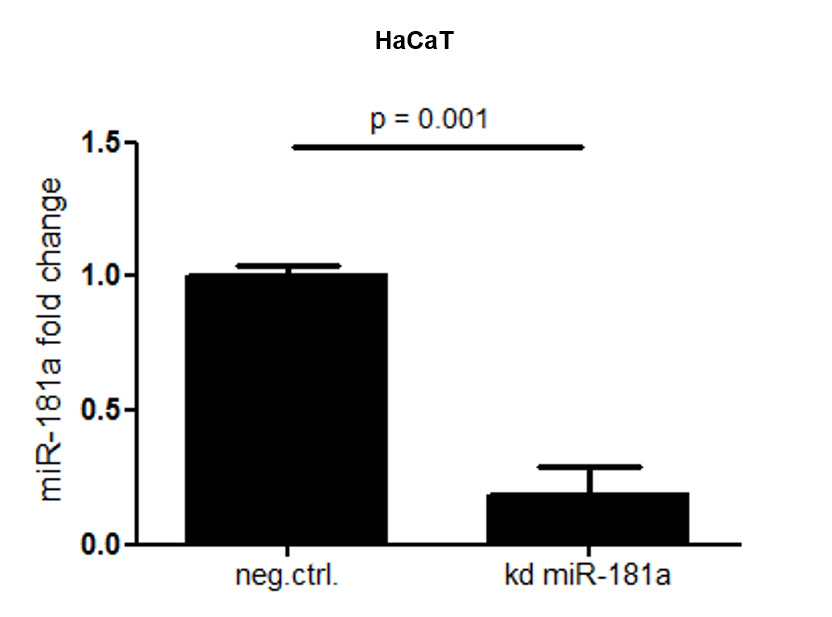

Supplement: S5 Fig — Whole RNA was isolated and transcribed into cDNA. miR-181a levels of HaCaT kd miR-181a were determined via TaqMan qPCR. Statistics were performed using Student’s t-Test. kd = knock down. (TIF) [file pone.0185028.s005.tif]

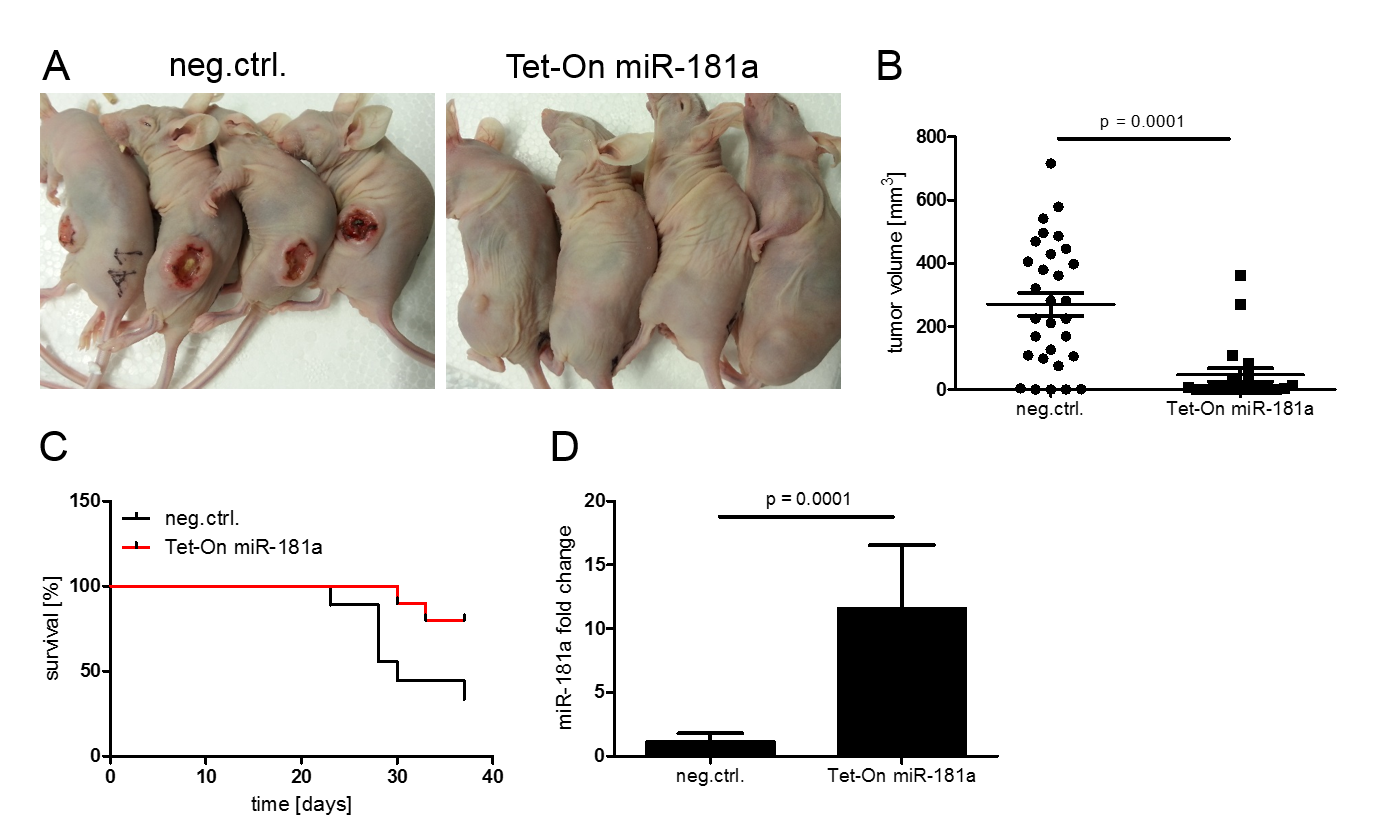

Supplement: S6 Fig — (A) SCC13 Tet-ON miR-181a were injected subcutaneously into nude mice. Doxycycline (200 mg/kg) was administered via food pellets. (B) Tumor volumes at the statistical endpoint of the experiment (day 21). (C) Kaplan-Meier survival curve. (D) miR-181a levels of tumors isolated after mice were terminated. Whole RNA was isolated and transcribed into cDNA follow by TaqMan qPCR. Statistics were performed using Student’s t-Test and Welch’s correction. (TIF) [file pone.0185028.s006.tif]

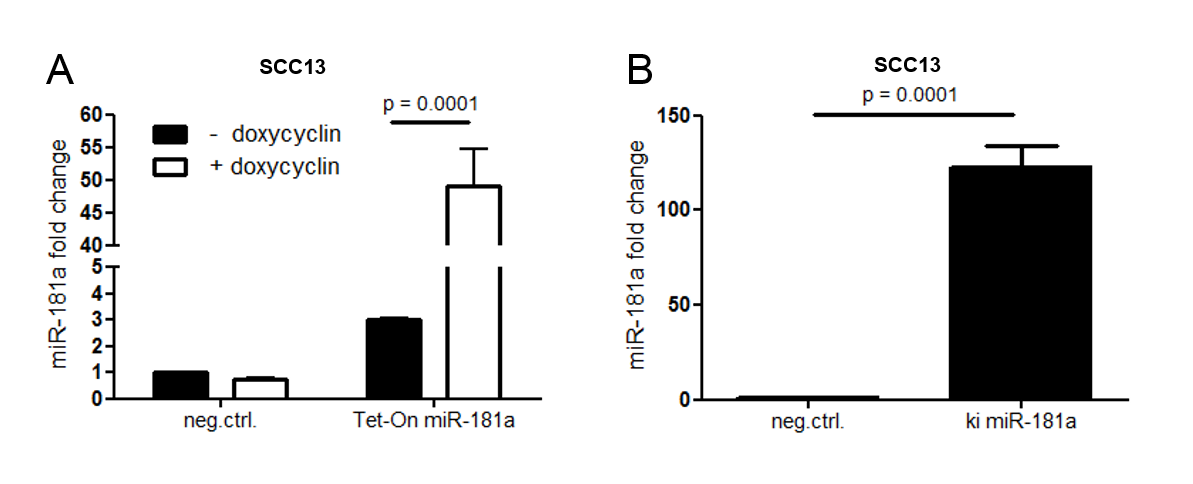

Supplement: S7 Fig — (A) SCC13 Tet-On miR-181a were incubated with 500nM doxycycline for 48 hours. (B) SCC13 stably over expressing miR-181a. Whole RNA was isolated and transcribed into cDNA followed by TaqMan qPCR. Statistics were performed using Student’s t-Test. ki = knock in. (TIF) [file pone.0185028.s007.tif]

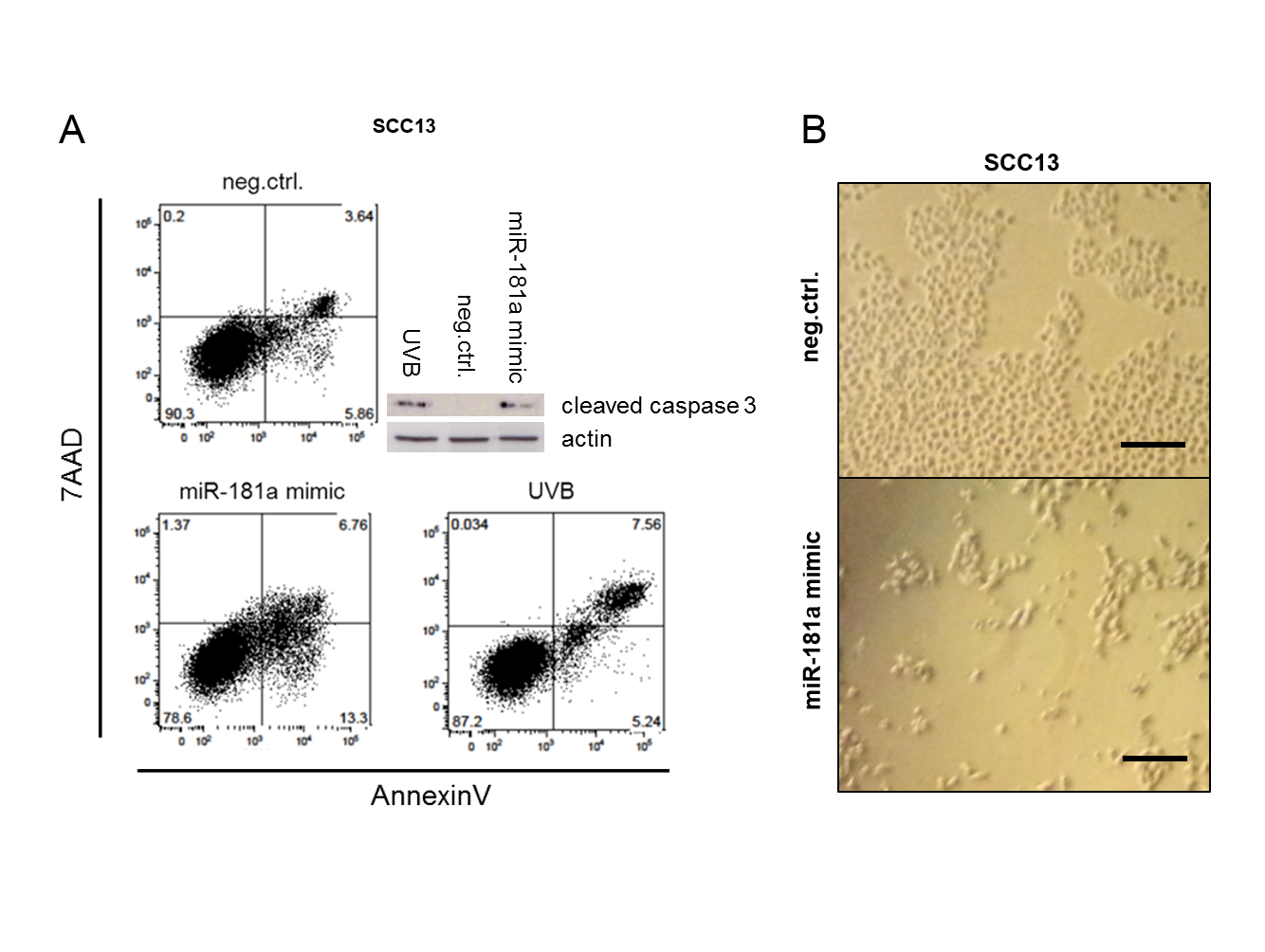

Supplement: S8 Fig — (A) SCC13 cells were transfected with miR-181a mimics for 48 hours. One fraction was stained for 7AAD and AnnexinV following FACS analysis. Cells were irradiated with 0.06 J/cm2 UVB for gating setup and served as a positive control. Protein from the other fraction was isolated and used for cleaved caspase 3 determination via Western blotting. (B). Cells were transfected with miR-181a mimics for 48 hours and seeded into petri dishes. 96 hours post transfection pictures were taken. Length of black bars = 150μm. (TIF) [file pone.0185028.s008.tif]

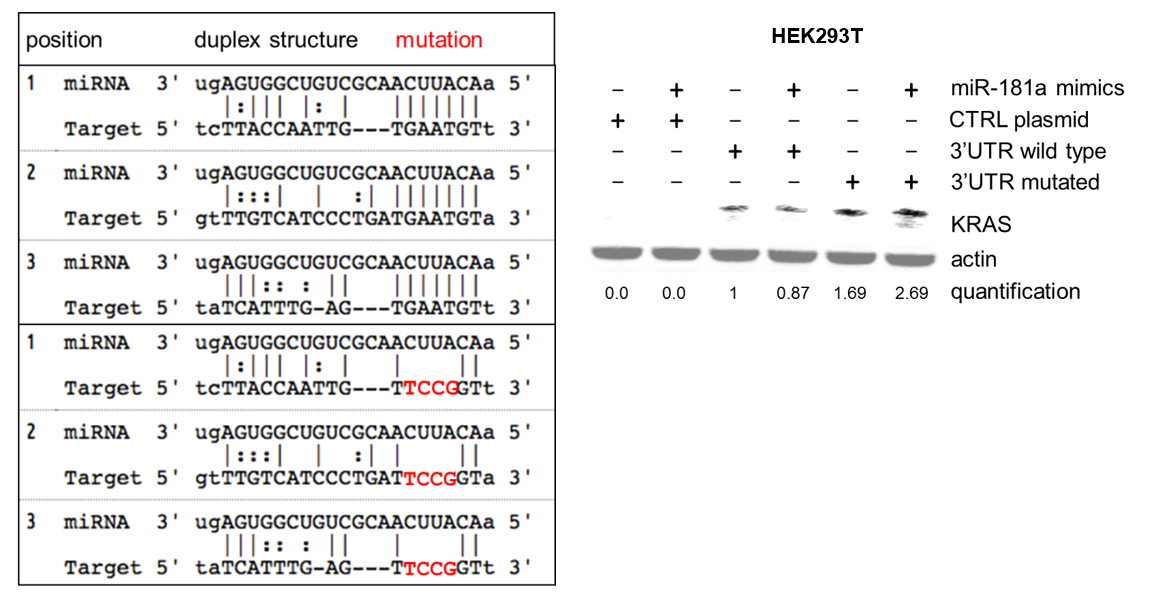

Supplement: S9 Fig — Indicated pUNO KRAS over expression plasmids and miR-181a mimics were transfected simultaneously into HEK293T cells for 48 hours. Protein was extracted followed by Western Blot. The panel in the left illustrates the three main miR-181a binding sites in KRAS 3’UTR and the according mutations. (TIF) [file pone.0185028.s009.tif]

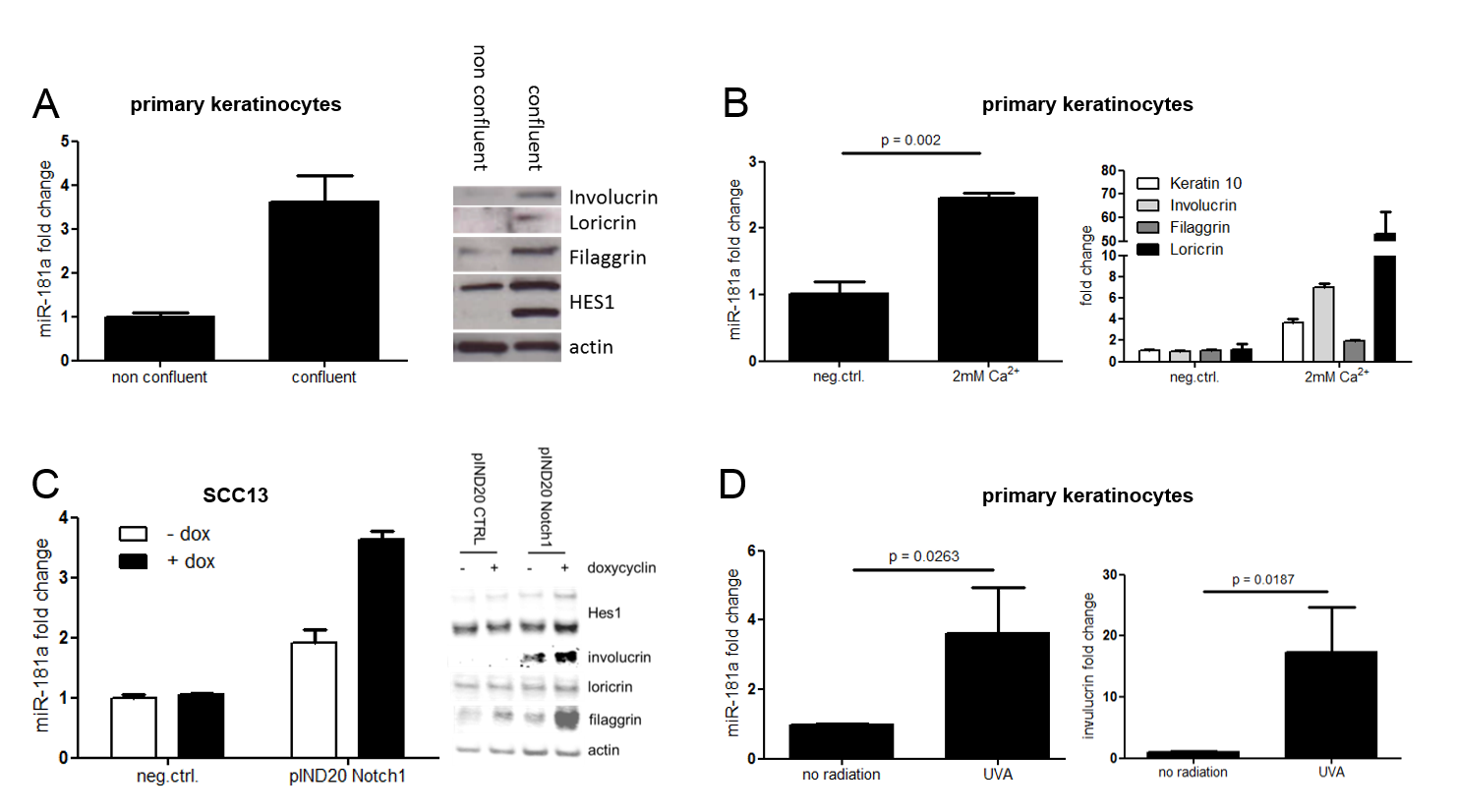

Supplement: S10 Fig — Cells were differentiated by keeping them in a confluent state for a week (A), by Ca2+ exposure (B), by transducing an inducible Notch1 plasmid (C) or by exposing them to 25 joule UVA irradiation. Protein and mRNA levels were determined by Western Blot or qPCR respectively. Student’s t-Test was used to calculate P values. (TIF) [file pone.0185028.s010.tif]

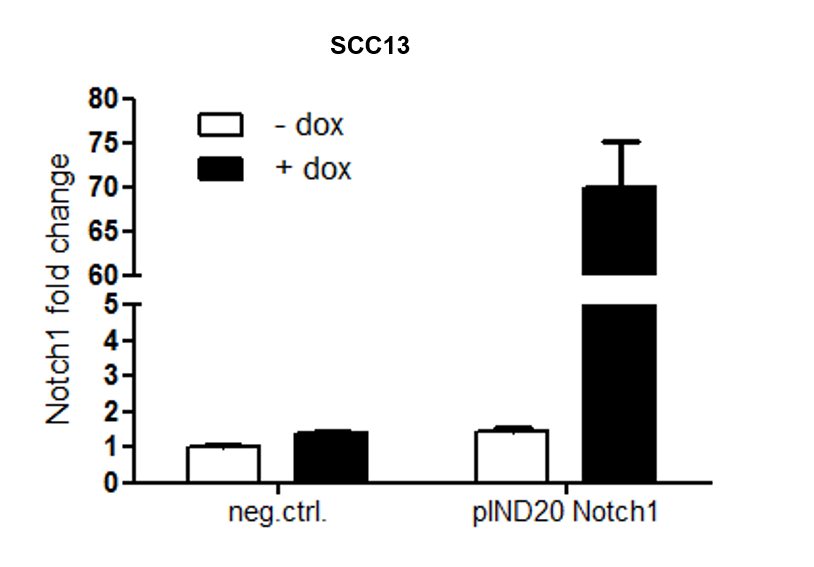

Supplement: S11 Fig — SCC13 pIND20 Notch1 were incubated with 500nM doxycycline for 48 hours. Whole RNA was isolated and transcribed into cDNA followed by TaqMan qPCR. qPCR primers were designed to target the expression sequence (intra-cellular domain of Notch1) of pIND20 Notch1. (TIF) [file pone.0185028.s011.tif]

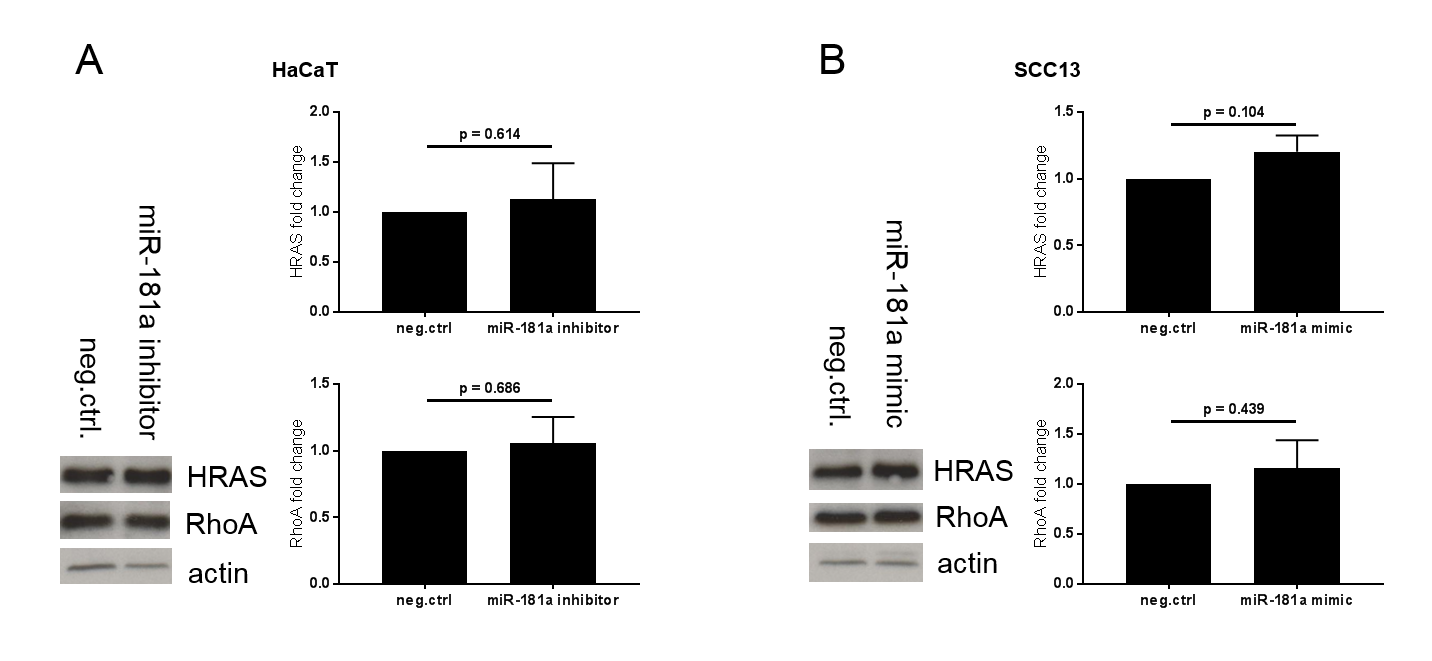

Supplement: S12 Fig — Cells were transfected with miRNA inhibitors (A) or mimics (B) for 48 hours. Cells were lysed and protein level analyses were performed using Western Blot. Total RNA extracts were used for oligo dT cDNA synthesis followed by SYBR Green qPCR. Error bars represent standard deviation and Student’s T test was used to calculate P values. (TIF) [file pone.0185028.s012.tif]
